# Perspectives of Family Caregiving Across Micro, Meso and Macro Levels

**DOI:** 10.1093/geroni/igaf122.716

**Published:** 2025-12-31

**Authors:** Hsueh-Fen Kao, Patricia Heyn

## Abstract

Family caregivers provide essential support to older adults, addressing both their own and the care recipient’s bio-psycho-social needs. While they play a vital role in promoting older adults’ well-being, their own health often suffers, leading to their recognition as ‘invisible patients.’ As the U.S. population rapidly ages and the healthcare system increasingly shifts toward community-based settings, family caregiving demands continue to rise, especially among minoritized populations who rely heavily on family support. These challenges extend beyond individual families (micro level) to communities and societal structures (meso level) and national policies, highlighting the need for systemic solutions (macro level). This symposium presents diverse perspectives on family caregiving across multiple health disciplines, including nursing, aging research, health sciences, and counseling. It begins with a personal journey illustrating the use of digital communication tools for long-distance caregiving. Next, It shares insights from long-term women caregivers of Mexican origin, detailing their experiences and the need to refine research protocols. The symposium also highlights two interventions for Alzheimer’s disease and related dementias: a web-based meaning-centered psychotherapy platform designed to enhance caregivers’ sense of purpose and reduce their loneliness, and a community-based wellness-focused initiative supporting Hispanic/Latino caregivers through the ‘health and wellness passport’ program to support caregivers who often report subjective cognitive decline and promote their well-being. Through these discussions, we aim to enhance understanding, encourage dialogue, and promote the well-being of at-risk caregivers.

